# The Stapes of Gomphodont Cynodonts: Insights into the Middle Ear Structure of Non-Mammaliaform Cynodonts

**DOI:** 10.1371/journal.pone.0131174

**Published:** 2015-07-15

**Authors:** Leandro C. Gaetano, Fernando Abdala

**Affiliations:** 1 Instituto de Estudios Andinos “Don Pablo Groeber”, Departamento de Ciencias Geológicas, Facultad de Ciencias Exactas y Naturales, Universidad de Buenos Aires, Ciudad Autónoma de Buenos Aires, Argentina; 2 Evolutionary Studies Institute, University of the Witwatersrand, Johannesburg, South Africa; 3 NRF/DST Centre of Excellence in Palaeosciences, Johannesburg, South Africa; The National Orchid Conservation Center of China; The Orchid Conservation & Research Center of Shenzhen, CHINA

## Abstract

The stapes is known in several non-mammaliaform cynodonts, although it has only been cursorily studied. Here we thoroughly analyze the stapedial anatomy of several basal cynodonts in a phylogenetic framework. Our study shows that the stapedial anatomy is more variable than previously thought. The morphological variation of the stapes led to the recognition of 11 phylogenetic characters that were included in a total evidence data matrix centered in the analysis of gomphodont cynodonts. Stapes morphology does not provide evidence to suggest a direct connection between the stapes and a postquadrate tympanic membrane (if present) and the hypothesis of a dorsal process as the site of attachment of a small ligament or the stapedial muscle is supported. The re-evaluation of the theories concerning the position of the tympanic membrane in non-mammaliaform cynodonts allowed us to conclude that the hypothetical postquadrate tympanic membrane associated with the squamosal sulcus is at best relictual and most likely non-functional (not connected with the stapes). The sound waves were most likely transmitted to the stapes from a postdentary tympanic membrane through the quadrate. Our analysis results in a better understanding of the auditory system in basal cynodonts and its evolution, highlighting the variability of the stapedial anatomy.

## Introduction

Mammals are the only extant representatives of the Synapsida, a more inclusive and morphologically diverse clade that comprises numerous fossil lineages [1]. Among them, non-mammaliaform cynodonts are a paraphyletic array of successively more crownward taxa that includes the sister-clade of mammaliaforms [2]. Non-mammaliaform cynodonts are known since the Late Permian and are particularly well represented in the Triassic of Gondwana and the Jurassic and Early Cretaceous of Laurasia [1, 3]. These taxa are profusely and exquisitely represented in the fossil record and constitute a very rich source of evidence to understand the origin of key mammalian features [1, 4].

Stepwise character modification in the fossil record is scarcely documented. The changes in the postdentary bones registered in the synapsid lineage constitute one of the best examples of gradual evolution of complex systems. Several taxa, particularly non-mammaliaform cynodonts and early mammaliaforms, depict morphological traits in the lower jaw and suspensorium that are successively closer to the definitive mammalian middle ear [5–6]. The evidence provided by basal synapsids support the Reichert-Gaupp Theory [7–8], which is mainly based on embryological data that suggest that the mammalian middle ear is ontogenetically derived from bones otherwise associated with the suspensorium [6, 9]. The mammalian middle ear includes three bones that link the tympanic membrane with the fenestra ovalis. In these forms, the middle ear structure allows effective transmission of sound through the air and reduces greatly the energy loss associated with the transition of sound waves from a low-density medium (air) to a high-density one (endolymph). The three bones in the middle ear (stapes, incus, and malleus) have been recognized as homologous to the columella auris, quadrate, articular, and goniale (prearticular) [10–14]. The columella auris or stapes is the only middle ear bone of non-mammalian tetrapods whereas the quadrate and the articular are involved in the suspensorium [15]. In synapsids, postdentary bones show gradual reduction and, especially among eucynodonts, a progressive detachment from the dentary, which constitutes the only lower jaw bone in advanced forms [15–17]. Non-mammaliaform cynodonts are especially relevant to understand the final changes that led to the mammalian auditory condition. These forms show notable reduction of the postdentary bones and the presence of a more delicate stapes [5–6]. The morphology of the postdentary bones of many non-mammaliaform cynodonts has been described exhaustively [5, 18–26]. On the other hand, few contributions analyze the stapedial anatomy of these taxa in detail and explore its morphological variations [5, 25, 27–30]. Only Novacek and Wyss [31] presented a comparative illustration of non-mammaliaform cynodont stapes, but they only discussed the general shape and the development of the stapedial foramen in the context of the morphology of stapes in living mammals.

Despite its light and fragile structure that makes it prone to postmortem damage or loss, the stapes has been recorded in many non-mammaliaform cynodonts. In the present contribution, the stapedial anatomy of several gomphodont cynodonts (*Diademodon tetragonus*, *Langbergia modisei*, *Trirachodon berryi*, *Scalenodon angustifrons*, *Luangwa drysdalli*, *Massetognathus pascuali*, *Menadon besairei*, and *Exaeretodon argentinus*) is surveyed for the first time in a comparative and phylogenetic framework. The availability of more than one specimen preserving the stapes of some of these taxa allowed for the analysis of intraspecific variability. Additionally, our analysis sheds light on the competing hypotheses regarding the position and characteristics of the tympanic membrane in non-mammaliaform cynodonts that have often relied on the poorly known stapedial anatomy [6].

## Materials and Methods

Stapes preserved in skulls of different gomphodont taxa were studied. The analyzed specimens are hold in the collections of the following institutions: Albany Museum, Grahamstown, South Africa (AM); Evolutionary Studies Institute (formerly Bernard Price Institute for Palaeontological Research), University of the Witwatersrand, Johannesburg, South Africa (BP); Council for Geosciences, Pretoria, South Africa (CGP); Field Museum of Natural History, Chicago, U.S.A. (FMNH); Geological Survey, Windhoek, Namibia (GSN); Museo Argentino de Ciencias Naturales Bernardino Rivadavia, Buenos Aires, Argentina (MACN); Museum of Comparative Zoology, Harvard University, Cambridge, U.S.A. (MCZ); Paleontologia de Vertebrados Lillo, Instituto Miguel Lillo, Tucumán, Argentina (PVL); Iziko South African Museum, Cape Town, South Africa (SAM); Antananarivo University, Madagascar (UA); and University Museum of Zoology, Cambridge, United Kingdom (UMCZ). The studied specimens include *Diademodon tetragonus*: BP/1/3773; *Langbergia modisei*: SAM-PK-K11481 and CGP 1/33; *Trirachodon berryi*: AM 461 and BP/1/4658; *Scalenodon angustifrons*: UMCZ T907; *Luangwa drysdalli*: GSN F 1527; *Massetognathus pascuali*: BP-1-4245, CRILAR PV 414, MCZ 3807, and PVL 4727; *Menadon besairei*: UA-10601; and *Exaeretodon argentinus*: MACN 18125, MCZ 4493, and MCZ 4510.

Some of these specimens are holotypes or have been included among the referred specimens in descriptive studies (SAM-PK-K11481, CGP 1/33, AM 461, BP/1/4658, UMCZ T907, UA-10601, MCZ 4493, MCZ 4510) [32–35]. Most of the remaining specimens (BP/1/3773, GSN F 1527, BP-1-4245, CRILAR PV 414, MCZ 3807, and PVL 4727) were taxonomically determined on the basis of the craniodental evidence as the stapes was associated to the skull.

Abdala and Smith [36] recognized the genus *Luangwa* in the upper Omingonde Formation; however, specific affinities were not proposed by these authors. Specimen GSN F 1527, found in the same formational levels, have been considered here as *Luangwa drysdalli* on the basis of some skull features and size.

*Massetognathus pascuali* is an abundant traversodontid cynodont from the Middle Triassic Chañares Formation of Argentina whose anatomy is relatively well known [37–40]. The presence of a snout subequal to temporal region and a well developed maxillary platform lateral to the postcanine row allowed for the assignation of the four specimens (BP-1-4245, CRILAR PV 414, MCZ 3807, and PVL 4727) to this species [35]. Additionally, BP/1/4245 and PVL 4727 show that upper postcanines lack an anterior cingulum, also a diagnostic feature of this taxon [35]. The smaller specimens, BP/1/4245 and PVL 4727, are interpreted as juveniles due to the relatively small skull size, the comparatively large orbits, and the zygomatic bars subparallel to the long axis of the skull in dorsal view.

Abdala et al. [33] identified specimen MACN 18125 as *Exaeretodon* sp. and commented on the morphology of the prootic, proatlas, and atlas as well as providing the skull basal length of this specimen. Nevertheless, these authors did not mention the presence of an associated stapes nor did they illustrate the specimen. Housed at the MACN collections, an isolated stapes was found together with two atlas arches and an atlas intercentrum in a box labeled as “Ischigualasto” and “MACN 18125”. The morphology and size of the stapes, the characteristics of the atlas centrum and arches, and the association and purported geological provenance (Ischigualasto Formation) of these elements make *Exaeretodon argentinus* the most parsimonious possibility and lead us to conclude that they belong to the individual numbered MACN 18125 that was analyzed by Abdala et al. [33].

For ease of expression and reference, a simple identifier for each specimen will be employed (Table 1). Each generic identifier consists of first four letters of the generic name, followed by a number to distinguish between specimens of the same taxon. When pertinent, a lower case “l” or “r” will be added to specify left or right stapes.

**Table 1:** Specimen identification key.

Taxon	Specimen number	Identifier
*Diademodon tetragonus*	BP/1/3773	DIAD1
*Langbergia modisei*	CGP 1/33	LANG1
	SAM-PK-11481	LANG2
*Trirachodon berryi*	AM 461	TRIR1
	BP/1/4658	TRIR2
*Scalenodon angustifrons*	UMCZ T907	SCAL1
*Luangwa drysdalli*	GSN F 1527	LUAN1
*Massetognathus pascuali*	BP/1/4245:	MASS1
	CRILAR-PV414	MASS2
	MCZ3807	MASS3
	PVL 4727	MASS4
*Menadon besairei*	UA-10601	MENA1
*Exaeretodon argentinus*	MACN 18125	EXAE1
	MCZ 4493	EXAE2
	MCZ 4510	EXAE3

## Description

The stapes of the analyzed taxa includes two crura (bicrurate condition) that converge together laterally and medially and define a stapedial foramen oriented dorsoventrally. The sector medial to the stapedial foramen includes the stapedial footplate which in turn was in contact with the fenestra ovalis. The sector lateral to the stapedial foramen is involved in the articulation with the quadrate. Either or both of these sectors may be extended into plate-like structures (thin dorsoventrally and relatively expanded lateromedially) for which the term “platform” is employed without implying a supporting role of any sort. The lateral and medial margins of the stapes can be alternatively projected anteriorly and/or posteriorly as seen in ventral or dorsal aspect. When present, a process emerging dorsally from the posterior crus is referred to as “dorsal process”.

### Diademodon tetragonus

Previous work–Parrington [28, 41] presented a stapes that he tentatively assigned to *Diademodon*. This author description was very brief, only commenting on the characteristics of the dorsal process and the size of the stapes and stapedial foramen. The specimen available to us is very similar to that illustrated by Parrington [41] suggesting co-generic affinities.

**Fig 1:**
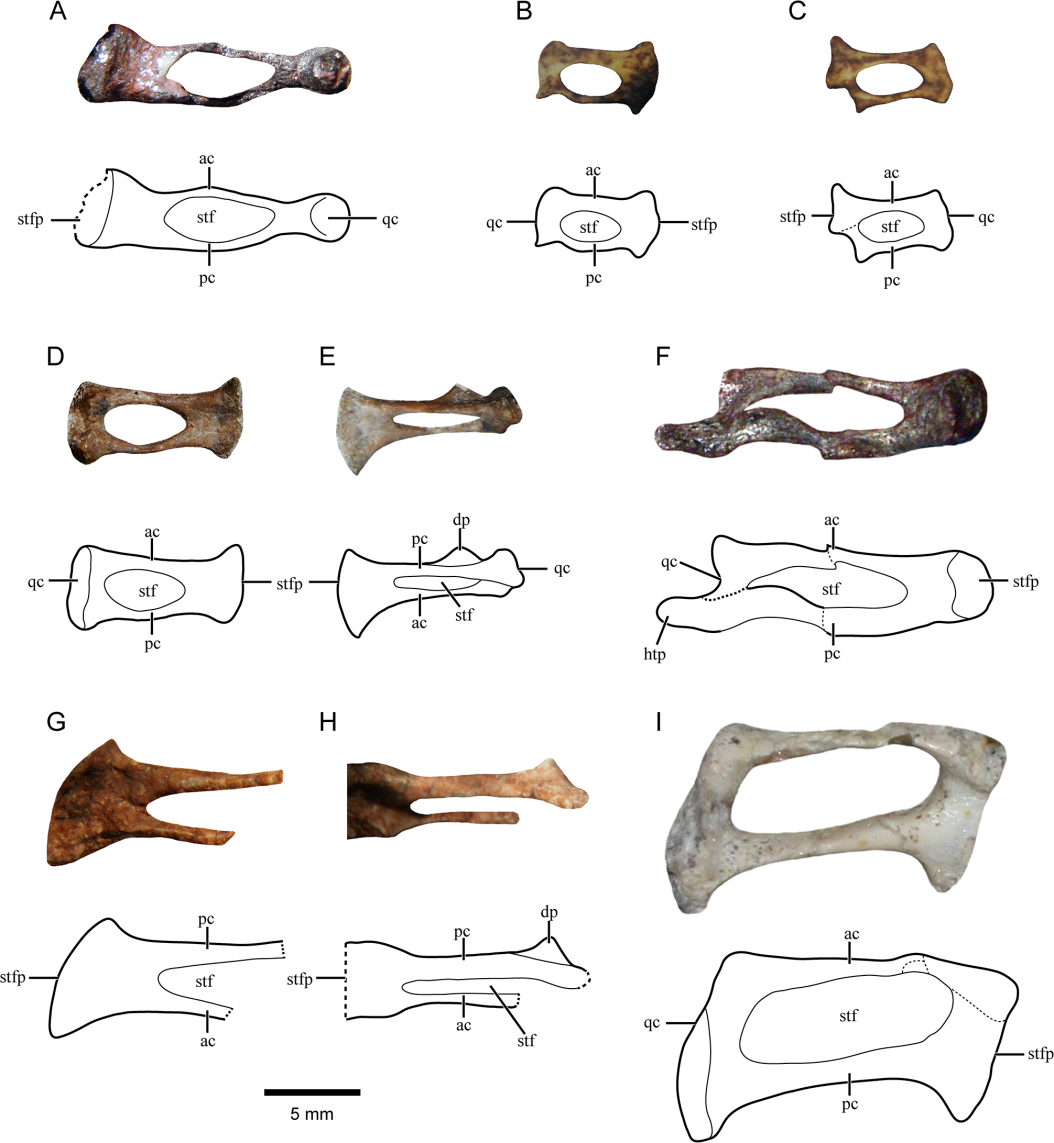
Gomphodont stapes. Photograph and line drawing of: A, *Diademodon tetragonus* (BP/1/3773, DIAD1) left stapes in ventral view; B-C, *Langbergia modisei* (SAM-PK-K11481, LANG2) right (A) and left (B) stapes in ventral view; D-E, *Trirachodon berryi* (BP/1/4658, TRIR2) right stapes in ventral (D) and posteroventral (E) views; F, *Scalenodon angustifrons* (UMCZ T907, SCAL1) left stapes in (mainly) dorsal view; G-H, *Luangwa drysdalli* (GSN F 1527, LUAN1) right stapes in ventral (G) and posteroventral (H) views; I, *Menadon besairei* (UA-10601, MENA1) right stapes in ventral view. Abbreviations: ac, anterior crus; dp, dorsal process; htp, hypothetical tympanic process or ossified extrastapes, interpreted here as an artefact due to deformation; pc, posterior crus; qc, quadrate contact area; stf; stapedial foramen; stfp, stapedial footplate. Dashed lines indicate fractures or incomplete margins. The 5mm scale bar applies to the whole figure.

Preservation–The two DIAD1 stapes are preserved in their original position. The general preservation is poor but the morphology can be ascertained (Fig 1A).

Anatomy–DIAD1 is slender and notably long with a triangular outline in ventral view, narrow laterally and broader medially. The anterior crus is wider anteroposteriorly than the posterior one (not recognizable in Fig 1A due to the poor preservation of the bone). The stapedial foramen is very long mediolaterally and relatively narrow anteroposteriorly. There is a broad medial platform showing a ventral concavity in its posterior half (Fig 1A). The stapedial footplate is circular and projected ventrally, also showing small anterior and posterior projections. Laterally, the crura converge into an anteroposteriorly narrow rod-like structure separated by a marked constriction from a spherical lateral end (Fig 1A). This rod-like structure represents the lateral platform observed in other cynodonts and is as long lateromedially as the medial platform. The narrow lateral portion of the stapes is in contact with the much broader medial surface of the quadrate trochlea. It is not possible to ascertain the presence of a dorsal process.

### Langbergia modisei

Preservation–LANG1 stapes is displaced with respect to the original position as the bone is ventro-posteriorly rotated and the fenestra ovalis is visible. The two LANG2 stapes are preserved in good condition (Fig 1B and 1C) but slightly displaced from their original position and a little rotated posteriorly. Additionally, LANG2r is displaced dorsally regarding it original position. It is noteworthy that both stapes are rotated similarly suggesting that their ultimate position is a result of the degradation of the surrounding soft tissues not the action of scavengers or physical agents.

Anatomy–LANG2 stapes has the anterior crus more robust than the posterior one. The stapedial foramen is ovoid and relatively small (almost 1/2 of the length of the bone). The lateral platform is relatively small (just the two convergent crura) whereas the medial platform is well developed and presents a ventral concavity. There are anterior and posterior projections medially and laterally (Fig 1B and 1C). The stapedial footplate does not seem to be projected ventrally. The lateral and medial margins of the stapes in ventral view are convex (Fig 1B). The dorsal process cannot be observed. In LANG1, the fenestra ovalis is exposed. It has the shape of an equilateral triangle with ventral and anterior borders well defined, unlike the dorsal one. The stapes is rotated with the larger posterior crus located ventrally to the anterior crus. The stapedial foramen is quite reduced due to deformation of the stapes. The medial platform appears to be more developed than the lateral one which is only formed by the union of the crura.

### Trirachodon berryi

Previous work–The stapes of *Trirachodon* was only described as a slender long rod of bone attached to the quadrate [42]. However, no illustration of this element or specimen number was provided. Subsequent mentions to the stapes of *Trirachodon* [27, 43] have been interpreted as taxonomic misidentifications [5].

Preservation–Only TRIR1r (holotype of *Trirachodon kannemeyeri*) is preserved whereas both stapes are present on TRIR2 close to their original position (Fig 1D and 1E). TRIR2r is almost perfectly preserved but only the anterior crus and parts of the medial and lateral platforms remain from TRIR2l.

Anatomy–*Trirachodon* stapes has an oval-shaped stapedial foramen. The lateromedial length of the stapedial foramen is more than half the length of the bone. The anterior crus is slightly more robust than the posterior one (Fig 1D). When compared to TRIR1, TRIR2 seems more slender with less curved and less robust crura. In TRIR2, the dorsal process is laminar with a triangular outline in posterior view which emerges dorsally from the posterior crus approximately in the central portion of the stapedial foramen and reaches the postero-lateral margin of the stapes (Fig 1E). A broad medial platform can be appreciated but, laterally, the crura converge into a very narrow platform (Fig 1D). The medial platform is almost twice the length of the lateral one. There is a posterior projection of the lateral margin of the stapes whereas the anterolateral corner is well rounded. A concavity is present in the ventral surface of the medial platform limited by the elevated continuation of the still recognizable shafts of the crura. The anteromedial corner is projected anteriorly as seen in ventral view (Fig 1D). The stapedial footplate is well ossified and longer antero-posteriorly than dorso-ventrally. The ventral border of the stapedial footplate is notched where both crura merge.

### Scalenodon angustifrons

Previous work–In his original description of *Scalenodon angustifrons*, Parrington [27] provided an illustration of the stapes and commented on its general morphology, focusing on the purported presence of an ossified extrastapes that became the center of much controversy (see below).

Preservation–SCAL1l (holotype) is displaced and damaged (Fig 1F). The whole bone as preserved has been rotated anteriorly. Additionally, except for the distal half of the posterior crus, it was flipped so most of the exposed surface is the dorsal side.

Anatomy–The crura are slender and subequal in size in ventral view (contra Parrington [27] who stated that the anterior crus was wider anteroposteriorly than the posterior one). Parrington [27] added that the anterior crus is “deeper” (dorsoventrally) when compared to the posterior one; this fact could not be ratified in the present study. Unlike the medial platform, the lateral platform is poorly developed. The stapedial foramen is large and elongated (approximately 2/3 of the length of the bone) but half of the opening is deformed (Fig 1F). The relatively small stapedial footplate is medially concave and oval in shape. There is a somewhat expanded projection laterally that seems continuous with the posterior crus (Fig 1F). However, the extension and characteristics of this structure cannot be confidently ascertained. Considering that such a process is unknown in any other non-mammaliaform cynodont, we interpreted this structure as an artifact due to postmortem deformation of the distal portion of the stapes, which is clearly evidenced by the deformation of the stapedial foramen.

### Luangwa drysdalli

Preservation–Both stapes are preserved in LUAN1. The left one is incompletely exposed and broken. The right one is close to the original position but pushed dorsally towards the skull. The lateral portion of the right stapes is missing but as it is the better preserved, it will be the base for the description below (Fig 1G and 1H).

Anatomy–The total length of the stapes can be ascertained due to the preservation of a minute portion of the posterolateral corner. The lateral platform is almost non-existent whereas the medial one is well developed (Fig 1G). The stapedial foramen is ovoid and large. The anterior crus is much thinner than the posterior one (Fig 1H). The stapedial footplate is not expanded ventrally. There are not ridges or depressions on the ventral surface of the medial platform. A posterior projection is present medially but not laterally (Fig 1G). Anterior projections of the medial and lateral margins cannot be ascertained due to poor preservation. A laminar, well developed dorsal process is present on the posterior crus medial to the lateral platform (Fig 1H). It points dorsally and has a triangular acuminate outline, being very similar morphologically to that of MASS1 (see below).

### Massetognathus pascuali

**Fig 2:**
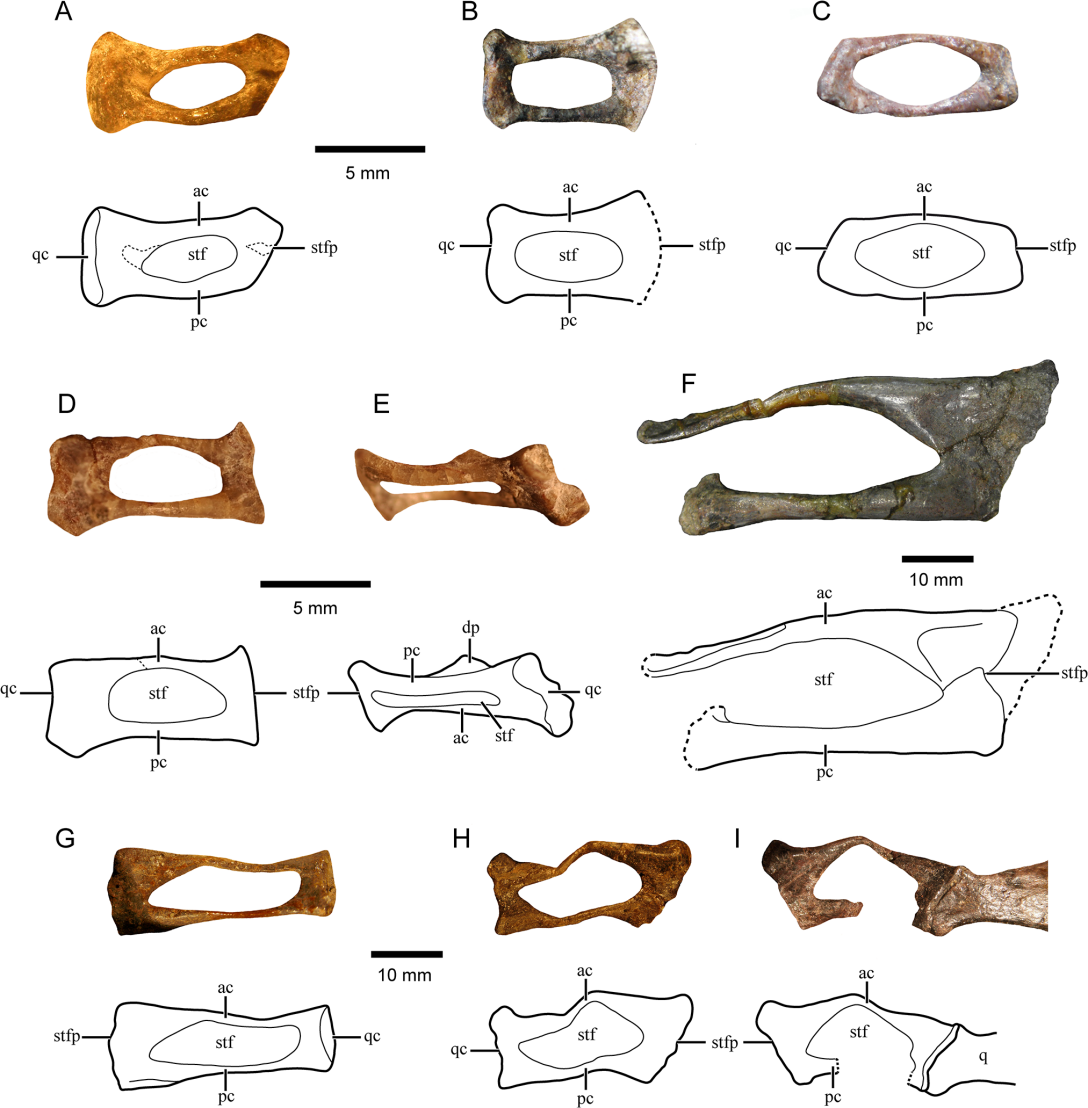
Gomphodont stapes. Photograph and line drawing of: A-E, right stapes of *Massetognathus pascuali* specimen MCZ 3807 [MASS3] (A), PVL 4727 [MASS4] (B), BP/1/4245 [MASS1] (C), and CRILAR PV 414 [MASS2] (D-E) in ventral (A-D) and posteroventral (E) views; F-I, *Exaeretodon argentinus* specimen MACN 18125 (EXAE1) left stapes (F), specimen MCZ 4510 (EXAE3) right stapes (G), and specimen MCZ 4493 (EXAE2) right (H) and left (I) stapes in ventral view. Abbreviations: ac, anterior crus; dp, dorsal process; pc, posterior crus; q, quadrate; qc, quadrate contact area; stf; stapedial foramen; stfp, stapedial footplate. Dashed lines indicate fractures or incomplete margins. The 5mm scale bar applies to A-E and the 10mm scale bar to F-I.

Preservation–MASS3r (Fig 2A) is preserved close to its original position but none of the natural contacts with the surrounding bones are recognized. Although some deformation might have affected the stapes, it is not evident and we regard the observable morphology as mainly unaltered. In MASS4, only the right stapes is present (Fig 2B). This element is probably almost in its original position although the quadrate is not preserved. MASS1 bears both stapes; the right one (Fig 2C) is undistorted whereas the left one seems to be deformed and with part of the lateral portion not preserved. The right stapes has been recovered in MASS2 (Fig 2D and 2E). Despite the skull has suffered strong compression, the stapes remains close to its original position. Nevertheless, the natural contacts between the stapes and the surrounding bones have been altered. The anterior crus is broken and its proximal half slightly rotated; the posterior crus is better preserved.

Anatomy–The stapes is composed by two slender crura defining an ellipsoidal stapedial foramen. These few general traits are the only ones shared by the four specimens studied.

The stapes is a slender bone in MASS1, stouter in MASS4, and having an intermediate condition in MASS2 and MASS3 (Table 2; Fig 2A–2E). In MASS2, the crura are slender and almost straight, parallel to each other (Fig 2D and 2E), whereas in MASS1 and MASS3, the crura are more robust and curved (Fig 2A and 2C). In MASS4, the crura are more robust than in any of the other specimens (Fig 2B) but straight as in MASS2. In MASS1 and MASS2, the anterior crus is less robust than the posterior one (Fig 2C–2E); however, there is a more accentuated size difference between the crura in MASS1 than in MASS2. On the other hand, the anterior crus is larger than the posterior one in MASS4 (Fig 2B) whereas the crura are subequal in MASS3 (Fig 2A). A particular feature of the crura in MASS3 is that they are notably developed dorsoventrally and therefore, they are not cylindrical but more extended dorsoventrally than anteroposteriorly. The stapedial foramen in MASS2 and MASS3 is approximately half of the lateromedial length of the bone (Fig 2A and 2D) whereas in the other specimens, it is slightly larger (i.e., ~2/3 of the lateromedial length of the bone; Fig 2B and 2C). Medially, there is a horizontal platform that is followed by an anteroposteriorly elongated stapedial footplate. In MASS1 and MASS2, this platform is very narrow (Fig 2D; not evident in Fig 2C due to the angle in which the picture has been taken) whereas it is slightly more expanded in MASS3 and MASS4 (Fig 2A and 2B). Ventrally, the central region of the medial platform is somewhat depressed in MASS2, MASS3, and MASS4 but not in MASS1. In MASS2, there are small but conspicuous anterior and posterior projections of the medial sector (Fig 2D) which are absent in MASS1 and MASS3 (Fig 2A and 2C). In MASS4, the medial margin of the stapes is not enough exposed to confirm the presence of such projections. Only MASS2 shows well developed lateral platform and a central shallow depression (Fig 2D). In MASS2, MASS3, and MASS4, the lateral margin of the stapes is somewhat expanded in ventral view (Fig 2A, 2C and 2D). The oval lateral surface of the stapes is visible in MASS3 where the ventral margin is more projected laterally than the dorsal one. The lateral surface of the stapes is concave in MASS2, MASS3, and MASS4 whereas in MASS1 it is convex and lacks an expanded margin ventrally. In MASS2, MASS3, and MASS4, the lateral margin of the stapes is projected posteriorly being this structure very robust in MASS3 (Fig 2A). An anterior projection of the lateral margin is also present in MASS3 and MASS4 (Fig 2A and 2B) but no projections are observed in the lateral region of MASS1 (Fig 2C). A delicate, laminar, triangular-shaped dorsal process is present only in MASS1 and MASS2. The dorsal process of MASS2 is developed on the posterior crus approximately between the mid-shaft and the beginning of the lateral platform (Fig 2E) whereas in MASS1 it is more lateral in position and mediolaterally shorter. The dorsal process of these specimens also differs in general shape being less pointy in MASS2 and not so dorsally projected as in MASS1. Additionally, the process is oriented dorsomedially in MASS1 whereas only a slight medial orientation is recognized in MASS2. As preserved in these specimens, the process does not contact any of the surrounding bones and no evidence of muscular insertion or cartilaginous covering is observable. Neither of the *Massetognathus* specimens analyzed preserves the stapes in natural position. Nevertheless, from MASS1 and MASS2, it is clear that the stapes contacted the quadrate laterally and that there is no evidence of a direct contact between the stapes and the paroccipital process.

**Table 2:** Skull and stapes measurements.

			Stapes			
Taxon	Specimen	Skull basal length (mm)	Length (mm)	Width (mm)	Stapedial foramen length (mm)	Stapes to skull basal length
*Diademodon tetragonus*	DIAD1	~115	14,4	~3.3	5,5	13%
*Langbergia modisei*	LANG1	89	8,9	3,7	~4	10%
	LANG2	65	6	–	3,2	9%
*Trirachodon berryi*	TRIR2	100	9,1	2,9	4,5	9%
*Scalenodon angustifrons*	SCAL1	~130	14,8	5,3	10	11%
*Luangwa drysdalli*	LUAN1	134	12	~2.8	6,6	9%
*Massetognathus pascuali*	MASS1	90	9	3,9	5,72	10%
	MASS2	135	9,4	3,9	5,32	7%
	MASS3	133	9,1	3,6	4,82	7%
	MASS4	90	8	4	4,9	9%
*Menadon besairei*	MENA1	169	16,3	6,8	11,1	10%
*Exaeretodon argentinus*	EXAE1	330	–	19,1	30,1	–
	EXAE2	234	~27	~11	~17	12%
	EXAE3	~290	31,1	8,7	21,8	11%

### Menadon besairei

Preservation–MENA1r is preserved on natural position and only minor damage is observed (Fig 1I).

Anatomy–It is a delicate slender bone with two thin straight crura. The anterior crus is thinner than the posterior one (Fig 1I). The crura converge medially and laterally into narrow platforms, representing no more than the convergent crura. The medial platform is slightly more developed than the lateral one, is concave ventrally and has a posterior projection. The lateral platform is curved anteriorly and a projection is present posteriorly (Fig 1I). The stapedial footplate is elongated antero-posteriorly and does not extend ventrally surpassing the ventral surface of the medial platform. The stapedial foramen is ovoid and extremely large (Fig 1I). A dorsal process is absent.

### Exaeretodon argentinus

Previous work–The stapes of *Exaeretodon argentinus* was described by Bonaparte [43] on the basis of specimen MACN 18063. This author presented a schematic drawing of the left stapes with the reconstructed medial portion and of the posterior region of the skull with both stapes as preserved. Bonaparte [43] stated that the stapes of *Exaeretodon* is characterized by a large stapedial foramen and two thin crura (the anterior crus thinner than the posterior one) that join medially to form a curved stapedial footplate but are separated laterally to contact the quadrate independently. The specimens available to us differ from MACN 18063 in all the features mentioned except in the proportion of the stapedial foramen. Intraspecific variability would explain the differences recorded; however, it is striking that almost no variation is observed among the three specimens analyzed here. A re-evaluation of specimen MACN 18063 would help to clarify this issue but, regrettably, specimen MACN 18063 could not be found at the museum collections at the time of our visit. Other mentions to the stapes of *Exaeretodon* referred to its contact with the quadrate [5, 28]. Allin [5] illustrated the stapes of MCZ 4493 (EXAE2, under the number MCZ 335-58M) pointing out that it fully contacted the quadrate laterally, an observation that was central to the discussion regarding the presence of an extrastapes and the connection with a purported postquadrate tympanic membrane [5, 28].

Preservation–The relatively well preserved left stapes is recognized in EXAE3 (Fig 2G) whereas in EXAE2 although both stapes are present they are highly distorted (Fig 2H and 2I). EXAE1 is an isolated right stapes missing the lateral platform (Fig 2F). The crura are broken but deformation is not evident. Medially, the crura are in contact with a small piece of bone interpreted as a fragment of the otic region of the skull.

Anatomy–The crura are very slender although the posterior crus is slightly more robust than the anterior one (Fig 1F–1I). In EXAE3, the crura are straight and almost parallel to each other (Fig 2G). On the other hand, the posterior crus of the specimen EXAE1 is straight whereas the anterior one is curved (Fig 2F). The crura are relatively well developed dorsoventrally when observed in posterior view. The stapedial foramen is ovoid and very large, extending lateromedially along most of the length of the stapes (Fig 2F–2I). Laterally and medially, the crura converge into narrow platforms that represent approximately 1/5 of the total length of the stapes (Fig 2G–2I). The medial platform is flat ventrally in EXAE2 and EXAE3 whereas a ventral depression is present medially in EXAE1. A slight central depression is observed ventrally on the lateral platform of specimen EXAE2 but its artificial origin due to deformation cannot be ruled out. In ventral view, the medial and lateral margins of the stapes are concave, the latter being bulbous. There are no projections anteriomedially and posteromedially in EXAE3 (Fig 2G) but an anteromedial projection is recognized in EXAE2 (Fig 2H and 2I). Laterally, anterior and posterior projections are observed only in EXAE2 (Fig 2H and 2I). There is no dorsal process on the posterior crus. Despite distortion, EXAE2l is preserved in natural contact with the quadrate showing that the whole lateral margin of the stapes contacted the quadrate (Fig 2I).

## Discussion

### Morphological variability

The non-mammaliaform cynodont stapes can be characterized as a bicrurate columnar bone perforated by a relatively large stapedial foramen which is dorso-ventrally oriented [31]. The reiteration of this general description in several contributions can give the false impression of a conserved morphology of the stapes among non-mammaliaform cynodonts. Our study however, shows that there is a high morphological variation represented in this tiny bone among different gomphodont taxa as well as intraspecifically.

Variations among different taxa result in a more or less robust stapes according to the predominance of the medial and lateral platforms or of a large stapedial foramen; in the development of a dorsal process on the posterior crus that can be present or absent and when present vary in relative position; in the different relative size of the crura which are similar to each other or one of them is larger; in the variable presence of projections in the extremes of the bone; and in the differentiation of flat or concave ventral areas in the medial and lateral platforms. *Diademodon tetragonus* represents the most clear departure from the “typical” condition observed in other gomphodonts showing an extremely long stapes with a triangular outline due to the reduction of the lateral side of the bone to a rod-like structure, a fact that results in the lateral portion of the stapes contacting a small portion of the comparatively larger internal quadrate trochlea.

Remarkable differences are depicted particularly in the stapes of *Massetognathus pascuali* with a stapedial foramen of different relative sizes, crura that are straight or curved, medial and lateral platforms with different degrees of development, anterior and posterior projections alternatively present, and a conspicuous dorsal process variably present and showing morphological differences among specimens as well as different relative placement on the posterior crura. Only the variation in the stapedial foramen size is correlated with the inferred ontogenetic stage of the specimens whereas the other variable traits highlight the morphological plasticity of the stapes intraspecifically. Opposite to this, the stapedial anatomy of *Exaeretodon argentinus* seems to be more regular intraspecifically.

Up to now, craniodental and postcranial features have been considered in the diagnosis and definition of basal cynodont taxa, but not the stapedial anatomy. The present study relied on the currently accepted diagnostic characters for the taxonomic identification of the taxa analyzed. In this scenario, the recognition of intraspecific variation of the stapes that cannot be alleged to different ontogenetic stages is surprising. Due to its direct impact on hearing capabilities, the stapes has always been considered as a morphologically conservative bone. Members of the same species are expected to be able to identify sounds of the same frequency range allowing for intraspecific communication as well as for perceiving the surroundings similarly. Under this scenario, our study points to the putative presence of cryptic species identified through differences in the stapedial anatomy. Nevertheless, the fact that the stapes is rarely preserved in specimens and the impossibility of testing the hearing capabilities of these taxa precludes the unambiguous identification of such species under variations only represented in this bone. Additionally, it is not possible to ascertain if the intraspecific variation in the stapedial anatomy is correlated with significantly different hearing capabilities. At present, we are unaware of the existence of studies analyzing the intraspecific variation of the stapedial anatomy in extant animals and its effects on audition that could be used as proxies to understand our findings in non-mammaliaform cynodonts.

### Phylogenetic implications

#### Character analysis

We present here an account of the variation recognized in the stapes among gomphodont cynodonts in a phylogenetic framework. The morphological variation of the stapes led to the recognition of 11 characters (discussed below) that we added to the data matrix of Melo et al. [44] (the character numeration corresponds to that of the data matrix; S1 Datamatrix).

Character 79: Number of crura: (0) one; (1) two.

Mono- and bicrurate stapes are known to be present in extant mammals [31]. Novaceck and Wyss [31] analyzed the significance of these different morphologies and pointed out that whereas the lack of a stapedial foramen is necessarily correlated with the stapedial artery not going through the stapes, the stapedial artery does not always pierce the stapes in bicrurate stapes. Additionally, these authors suggested that monocrurate stapes are thought to be of positive fitness for aquatic mammals whereas bicrurate stapes provide enhanced hearing abilities for terrestrial forms; however, it is important to note that the functional significance of these conditions has not been explored in detail comparing animals of similar habitats.

Character 80: Curvature of the crura: (0) both crura straight; (1) both crura curved; (2) posterior crus curved, anterior crus straight; (3) anterior crus curved, posterior crus straight.

There are no studies to our knowledge that evaluate the different curvature observed in the crura of extant or fossil cynodonts, its implications in sound conduction or its correlation to the stapedial artery characteristics. We tentatively propose here that the curvature of the crura might be related to the diameter of the stapedial artery relatively to that of the stapedial foramen. This hypothesis rests on the fact that the stapedial artery precedes the ossification of the stapes [31] and should await proper analyses in extant forms for more conclusive evidence. Although we acknowledge that it is not always the case in extant mammals [31], if the stapedial artery is reconstructed as piercing the stapes in the basal cynodonts analyzed here, then the crura would be expected to acquire curvature when the stapedial artery diameter exceeds the anteroposterior width of the stapes. Considering the plasticity of the stapes and stapedial artery during ontogeny registered in extant mammals [31], this scenario would explain the intraspecific variation of the crura curvature observed in *Massetognathus*.

Character 81: Relative size of the stapedial foramen: (0) large, ~3/4 of the total length of the stapes or more; (1) medium-sized, ~2/3 of the total length of the stapes; (2) small, ~1/2 of the total length of the stapes or less.

The relative size of the stapedial foramen can be interpreted as a measure of the robustness of the stapes: the larger the stapedial foramen, the lighter built the stapes. Lightening of the stapes seems to be in line with an increased hearing sensibility given the mass reduction of (at least) one of the sound transmitting elements [5, 26].

Character 82: Ossified platforms of the stapes: (0) medial and lateral ossified sectors restricted to the convergent crura; (1) only the medial ossified sector constituting a platform; (2) only the lateral ossified sector constituting a platform; (3) medial and lateral ossified sectors constituting platforms.

Medially and laterally, the crura converge delimiting the stapedial foramen. In some of the taxa analyzed, the crura continue into medial and/or lateral platforms that result in the reduction of the stapedial foramen size in relation to the length of the stapes. The absence of medial/lateral platforms, together with a relatively large stapedial foramen and slender crura, imply a more lightly stapes.

Character 83: Relative size of the ossified portion of the stapes medial and lateral to the stapedial foramen: (0) medial portion wider (lateromedially) than the lateral one; (1) lateral portion wider (lateromedially) than the medial one; (2) medial portion as wide (lateromedially) as the lateral one.

Regardless of the presence of medial/lateral platforms, the relative development of the ossified portion of the stapes medial and lateral to the stapedial foramen is variable among the taxa analyzed. Although, it is not possible to evaluate the functional implications of this variability, we find this character to be phylogenetically informative (see below).

Character 84: Ossified dorsal process of the stapes: (0) present; (1) absent.

The dorsal process of the stapes is a delicate flange-like laminar structure at the dorsal surface of the posterior crus pointing dorsally or dorsomedially. This process cannot be readily homologized with the tympanic process of other tetrapods. The lack of a direct connection between the stapes and the postquadrate tympanic membrane in cynodonts renders the functional implications of this process uncertain (see discussion below). In extant mammals, the stapedius is the only muscle attached to the stapes, connecting this bone to the wall of the tympanic cavity [6, 31, 45]. The dorsal process observed in non-mammaliaform cynodonts could have been the attachment site for a homologous muscle.

Character 85: Anterior projection of the medial margin of the stapes in ventral view: (0) absent; (1) present.

Character 86: Posterior projection of the medial margin of the stapes in ventral view: (0) absent; (1) present.

Character 87: Anterior projection of the lateral margin of the stapes in ventral view: (0) absent; (1) present.

Character 88: Posterior projection of the lateral margin of the stapes in ventral view: (0) absent; (1) present.

When analyzed in ventral aspect, anterior and/or posterior projections of the medial and/or lateral margins of the stapes are observed in some of the cynodonts studied. Comparisons regarding these features show a great amount of variation although some of the character states are recovered as synapomorphies in our analysis (see below).

Character 89: Stapedial footplate expanded dorsoventrally: (0) absent; (1) present.

Variations on the characteristics of the stapedial footplate might be important to understand the mechanical basis of sound transmission; however, such an analysis depends on detailed studies that only can be performed in extant forms and thus exceeds the scope of the present contribution.

Character 90: Stapes length relative to the skull basal length: (0) greater than 7%; (1) less than 5.5% (from Wible and Hopson, 1993).

Rowe [46] introduced the relative size of the stapes as a phylogenetic character; but Wible and Hopson [47] defined the character states quantitatively. They distinguished between taxa in which the stapes was greater than 7.5% and those in which it represented less than 5.5% of the total length of the skull [47]. Furthermore, they recovered the latter state as an unequivocal synapomorphy of a clade including *Sinoconodon*, Morganucodontidae, their most recent common ancestor, and all its descendants (“node 3” of Wible and Hopson [47]; Mammaliaformes of Rowe [46]).

#### Phylogenetic hypotheses

**Fig 3:**
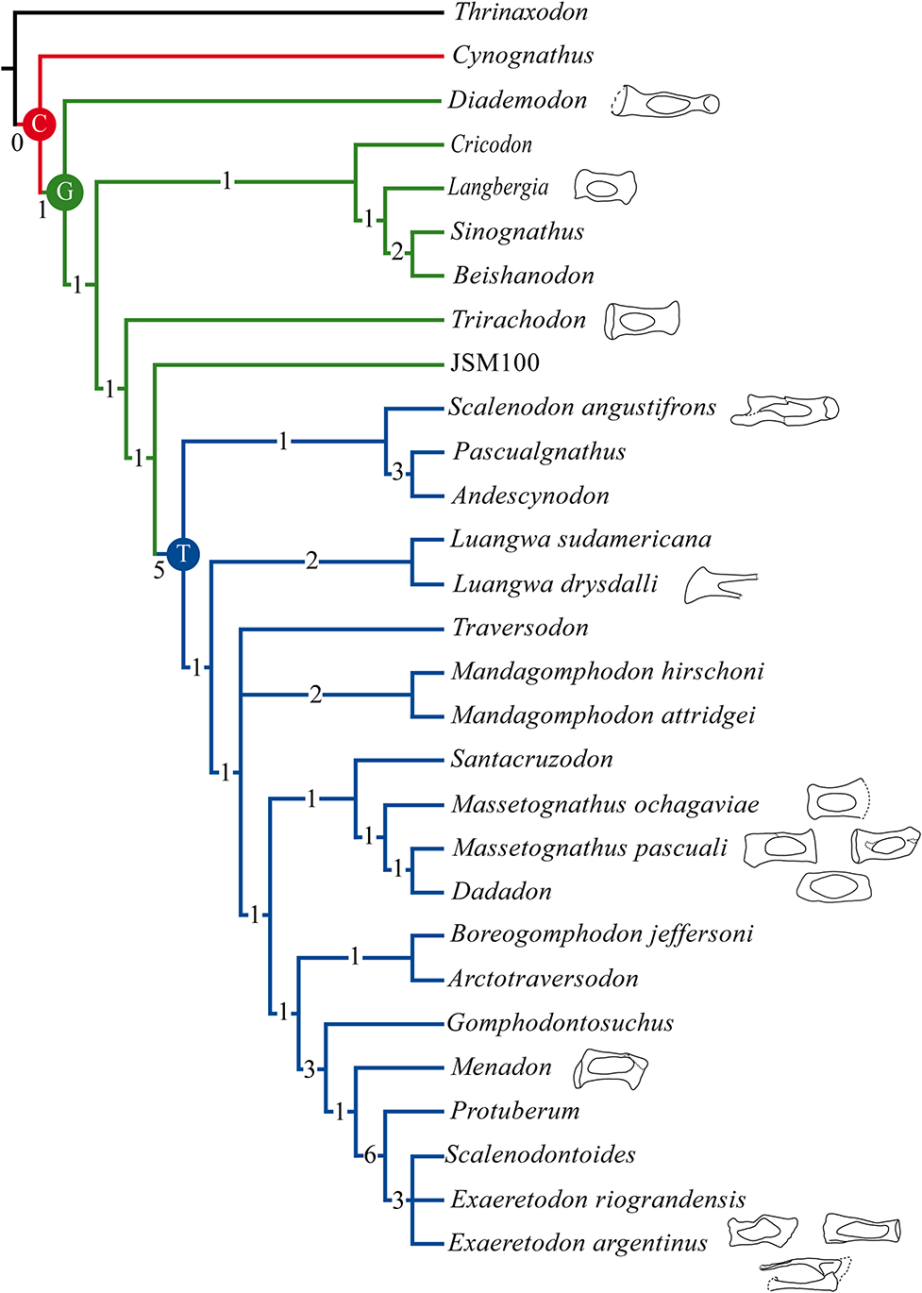
Phylogenetic analysis. Strict consensus tree of the two most parsimonious trees found in the analysis excluding *Nanogomphodon*. Numbers represent the Bremer support. Letters at the nodes indicate high-level clades: C, Cynognathia; G, Gomphodontia; T, Traversodontidae.

We included the recognized characters in a modified version of Liu and Abdala’s [35] data matrix centred on the phylogenetic relationships of Gomphodontia, presented by Melo et al. [44] who, among other minor changes in the codification, consider seven multistate characters (2, 10, 25, 31, 32, 48, and 73) as additive. The resultant data matrix of 90 characters and 30 taxa [S1 Datamatrix] was analyzed using TNT version 1.1 [48–49] [S2 Datamatrix]. We conducted a heuristic search starting from 100 random addition sequences and keeping up to 100 trees in memory followed by the application of the TBR algorithm. Our analysis rendered two most parsimonious trees of 235 steps with a consistence index (CI) of 0.472 and a retention index (RI) of 0.727 which only differ in the position of *Traversodon* [S1 Fig]. A second analysis in which *Nanogomphodon*, represented solely by an isolated lower postcanine, was excluded (see Melo et al. [44]), resulted in two optimal cladograms of 234 steps with an associated CI and RI of 0.474 and 0.729, respectively [S2 Fig]. Similar to the first analysis, the most parsimonious trees obtained are identical except for the position of *Traversodon* and the topology of the strict consensus remains well resolved (Fig 3). The strict consensus trees resulting from these analyses show only some minor differences with the study of Melo et al. [44]. In our analysis, as well as in that of Melo et al. [44] and in the majority-rule consensus tree of Liu and Abdala [35], *Nanogomphodon* is shown as the most basal traversodontid [S3 Fig]. Nevertheless, Liu and Abdala [35] interpreted this phylogenetic position to be spurious and due to the very incomplete representation of this genus, a suggestion that we believe justified.

The trirachodontids *Cricodon*, *Langbergia*, *Sinognathus*, and *Beishanodon* form a monophyletic grouping of basal gomphodonts whereas *Trirachodon* and JSM100 (a purported juvenile of *Trirachodon* sp. [35, 50]) are successive stems of Traversodontidae (after Kammerer et al. [51]) in both of our analyses. A non-monophyletic Trirachodontidae was also obtained in the strict consensus of the analyses presented by Melo et al. [44] (when including *Nanogomphodon*), Gao et al. [52], and Liu and Abdala [35]. The forced monophyly of Trirachodontidae in the present study including *Nanogomphodon* rendered 239 equally parsimonious trees two steps longer (237 steps) in which the phylogenetic relationships among traversodontids are poorly resolved [S4 Fig]. A similarly constrained analysis excluding *Nanogomphodon* yielded 13 optimal cladograms, only one step longer (235 steps) than the unconstrained searches (without this taxon) and differing only in the presence of a polytomy at the base of Traversodontidae between *Scalenodon angustifrons*, the clade formed by *Pascualgnathus* and *Andescynodon*, and the remaining traversodontids [S5 Fig]. The latter trichotomy is also observed in the results of Liu and Abdala [35] and Melo et al. [44]. On the other hand, regardless of the inclusion of *Nanogomphodon*, our unconstrained analyses yield a basal monophyletic grouping of traversodontids including *Scalenodon angustifrons* and the sister-taxa *Pascualgnathus* and *Andescynodon*.

The mapping of the stapedial characters in the strict consensus tree excluding *Nanogomphodon* obtained here (Fig 3) shows that some of them are phylogenetically informative whereas others highlight the morphological variability of this bone even intraspecificaly or among closely related taxa.

**Fig 4:**
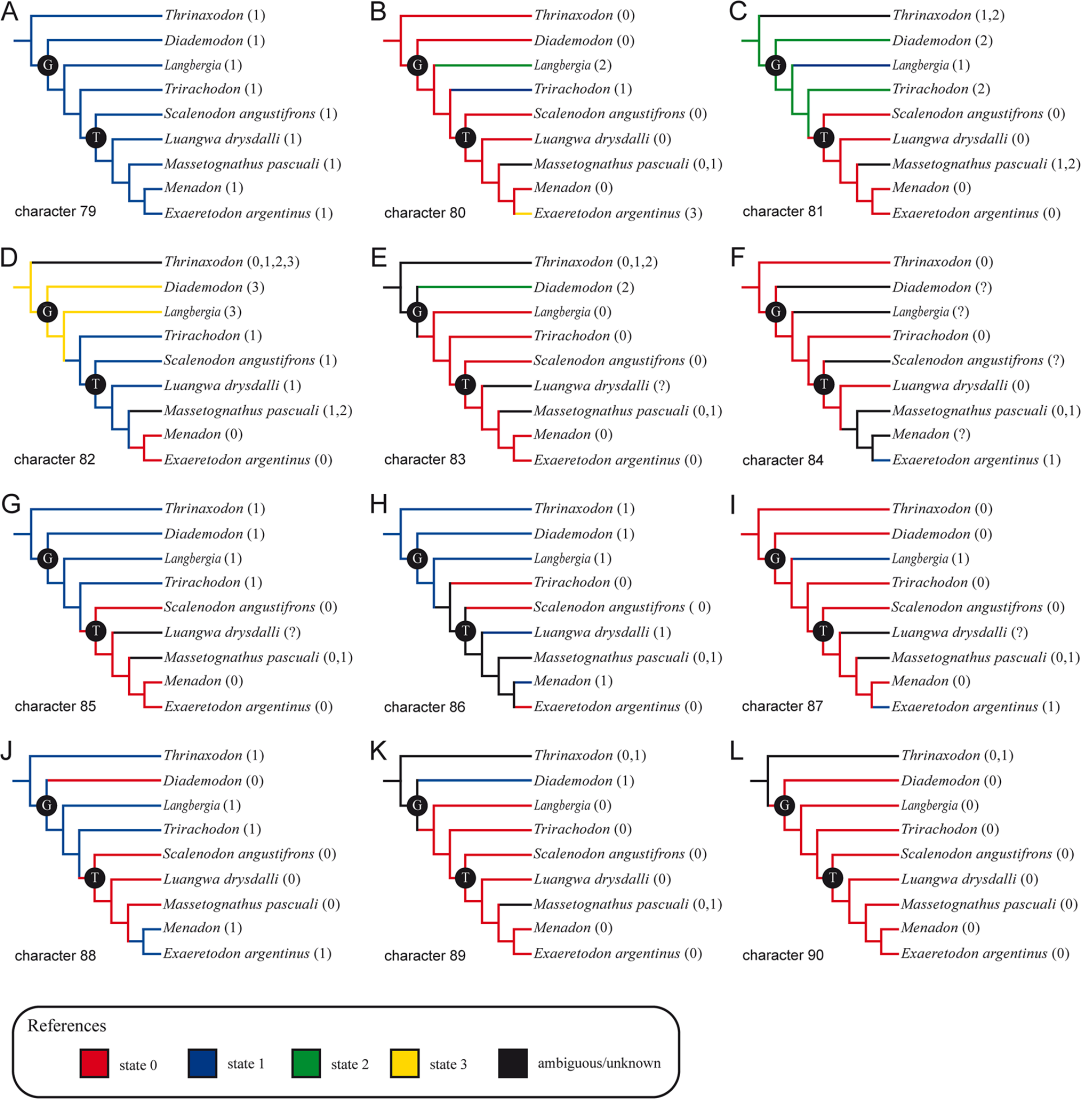
Stapedial character states distribution. Pruned strict consensus trees showing the states distribution of the stapedial characters 79 to 90 (A-L). Letters at the nodes indicate high-level clades: G, Gomphodontia; T, Traversodontidae. Numbers between brackets represent the codification for each taxon.

All gomphodonts have a bicurated stapes (character 79:1), a condition that seems to have been widespread among non-mamaliaform cynodonts (Fig 4A) and also observed in the early mammaliaforms *Morganucodon* [53] and *Haldanodon* [54]. A rod like imperforate stapes was suggested to be present in *Bienotherium* [55]. On the other hand, Novacek and Wyss [31] stated that according to Wible and Hopson (personal communication) the purported stapes of *Bienotherium* should be re-interpreted as a piece of the ossified hyoid arch. Although there is not enough preserved of this bone to assure its identification, the latter view seems more probable. Nevertheless, if it were interpreted as a stapes, the extremely poor preservation avoids the identification of any relevant anatomical trait and it cannot be ascertained if one or two crura were present.

The presence of straight crura (character 80:0) is the basal and more widespread condition for non-mammaliaform cynodonts (Fig 4B). Non-eucynodont cynodonts (*Procynosuchus*, *Galesaurus*, *Thrinaxodon*, and *Platycraniellus*) have straight parallel crura [56]. This condition is plesiomorphic for cynognathids and the more derived gomphodonts. Nevertheless, *Trirachodon* has both crura curved (character 80:1) and *Langbergia* possesses an intermediate condition with the posterior crus curved and the anterior crus straight (character 80:2). Most traversodontids (*Luangwa*, *Menadon*, *Scalenodon*, MASS2, and MASS4) share the presence of straight parallel crura (character 80:0). MASS1, similar to *Trirachodon*, has curved crura (character 80:1) whereas in *Exaeretodon* the anterior crus is curved and the posterior one straight (character 80:3).

Although not included among the characters analyzed here, the relative anteroposterior width of the crura in ventral view is variable among the taxa analyzed In MASS1, MASS2, and in the closely related traversodontids *Exaeretodon*, *Luangwa*, and *Menadon*, the posterior crus is more robust than the anterior one. In the trirachodontids *Langbergia* and *Trirachodon* as well as in MASS4, the anterior crus is relatively more robust than the posterior one. In *Scalenodon* and in MASS3, the crura are subequal.

In the traversodontids *Exaeretodon*, *Luangwa*, *Menadon*, and *Scalenodon*, the stapedial foramen is relatively large, its mediolateral length representing ~3/4 of the total length of the stapes. Thus, this feature (character 81:0) is recovered as a synapomorphy of the traversodontid clade (Fig 4C). Nevertheless, it is worth mentioning that this characteristic is highly variable intraspecifically in the traversodontid *Massetognathus*. The relatively smaller stapedial foramen of MASS2 is comparable to more basal taxa (e.g., *Diademodon* and *Trirachodon*), in which it is approximately half the length of the bone (character 81:2). In MASS1 and MASS4, the stapedial foramen/stapedial length ratio is ~2/3 (character 81:1), a condition also observed in the trirachodontid *Langbergia*.

The variable development of medial and/or lateral platforms is mapped in our most parsimonious trees as synapomorphic of several nodes (Fig 4D). The presence of medial and lateral platforms (character 82:3) is the basal condition for gomphodonts as observed in *Diademodon* and *Langbergia* (Fig 4D). The development of a medial platform (character 82:1) is recovered as a synapomorphy of the node including *Trirachodon* and traversodontids (Fig 4D). The absence of medial and lateral platforms (i.e., the medial and lateral ossified sectors are restricted to the convergent crura) is a synapomorphy of the clade including *Menadon* and *Exaeretodon* (character 82:0; Fig 4D). *Massetognathus* shows a remarkable intraspecific variation of this character. Like *Diademodon* and *Langbergia*, MASS3 has the medial and lateral platforms present (character 82:1) whereas MASS4 is comparable to *Trirachodon* and *Scalenodon* (medial platform developed; character 82:1), and MASS1 shares with *Menadon* and *Exaeretodon* the absence of medial and lateral platforms (character 82:0). In MASS2 only the lateral platform is present (character 82:2).

Irrespective of the development of medial and/or lateral platforms, the ossified portion of the stapes medial to the stapedial foramen (character 83: 0) is synapomorphic of the clade including trirachodontids and traversodontids (Fig 4E). MASS1 and MASS2 represent the exception among traversodontids as they present the lateral portion of the stapes wider lateromedially than the medial one (character 83: 1). In *Diademodon*, the ossified areas medial and lateral to the stapedial foramen have similar size (character 83: 2).

The presence of a dorsal process (character 84:0) is recognized in *Luangwa*, *Trirachodon*, and in MASS1 and MASS2 (Fig 4F) whereas it is absent in *Exaeretodon* (character 84:1). The evaluation of the dorsal process in other gomphodonts is not feasible.

The presence of projections medially and/or laterally has proved to be phylogenetically informative (Fig 4G–4J). The absence of an anterior projection medially (character 85:0) and of a posterior projection laterally (character 88:0) are recovered as synapomorphic of traversodontids (Fig 4G and 4J). Additionally, the reversion of this latter trait to the ancestral condition (character 88:1) is a synapomorphy of the clade including *Menadon* and *Exaeretodon* (Fig 4J).

The dorsoventrally little expanded stapedial footplate (character 89:0) is synapomorphic of the clade including trirachodontids and traversodontids (Fig 4K). Only in *Diademodon*, MASS1, and MASS3, the stapedial footplate is projected ventrally (character 89:1). The stapedial footplate is particularly small relative to the lateromedial length and anteroposterior width of the stapes in *Exaeretodon* and *Scalenodon*.

Gomphodonts retain a relatively large stapes when compared to the basal skull length (character 90:0), with ratios between stapes/skull basal length ranging from 7% (in the largest *Massetognathus* specimens) to 13% in *Diademodon* (Table 2; Fig 4L). Intraspecific variation of this feature is clearly observed among *Massetognathus* specimens in which the stapes length ranges between 7% and 10% of the basal skull length. In the remaining taxa analyzed, the stapes represented more than 9% of the basal skull length, and the intraspecific variation (observed in *Exaeretodon* and *Langbergia*) was never greater than 1%.

### Middle ear structure: the stapes and the tympanic membrane

The study of the stapes of non-mammaliaform cynodonts is of upmost importance to understand the evolution and origin of the mammalian middle ear. Previously, only the stapes of a few taxa have been described in detail. Most of the published work was aimed to the recognition of reptilian or mammalian characteristics on the stapes of non-mammaliaform cynodonts. The middle ear structure of non-mammaliaform cynodonts is derived from the monossicular condition present in more basal synapsids and certainly different from both the triossicular structure of early mammaliaforms and mammals and the monossicular pattern of living reptiles [5–6, 9, 14–15, 27, 57–61].

**Fig 5:**
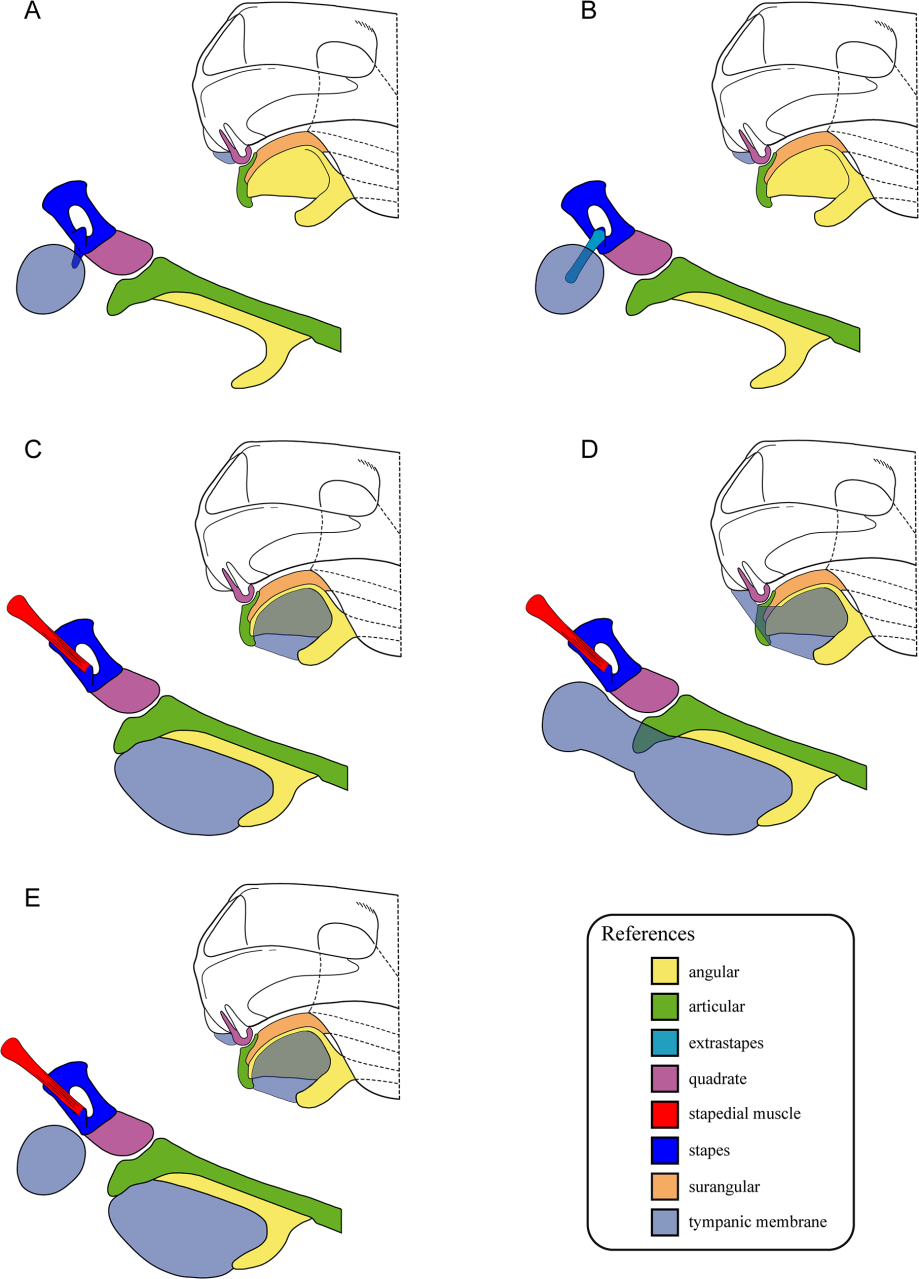
Schematic reconstructions of the posterior portion of the skull and lower jaw in lateral view showing different hypotheses regarding the stapes and tympanic membrane. Diagrams depicting: A-B, a single postquadrate tympanic membrane at the inner end of the squamosal sulcus; C, a single tympanic membrane at the postdentary portion of the lower jaw; D a single tympanic membrane continuous from the postdentary portion of the lower jaw to the postquadrate region; E, two separate tympanic membranes at the postdentary and postquadrate regions. The stapes is represented with the lateral surface fully contacting the quadrate and bearing a tympanic process (A), an extrastapedial portion attached to the dorsal process (B) or with the stapedial muscle attached to the dorsal process (C-E). Modified from [63] and [66].

One of the major issues regarding the middle ear of non-mammaliaform cynodonts is the position of the tympanic membrane. Although Kemp [26] introduced a reasonable argument against an air-filled cavity (i.e., tympanic cavity) in non-mammaliaform cynodonts, posing a linguistic problem to the recognition of a tympanic membrane which is defined as a membrane bounding the tympanic cavity; for the purpose of the present discussion, we will refer to the specialized area of acoustically isolated tissue that would have acted as an air-borne sound receiver in non-mammaliaform cynodonts [26] as ‘tympanic membrane’. Allin and Hopson [6] presented the most thorough up-to-date review of the different theories that have been proposed regarding this subject (see also Meng et al. [62]); thus, an exhaustive recapitulation of those hypotheses will not be repeated here. However, for the purpose of the present discussion and considering the new information available, we believe it is important to analyze some aspects of the three theories that have been dominant. The first one, principally supported by Westoll [57, 63], Parrington [27–28, 64], and Hopson [65], considers that the tympanic membrane was associated with the squamosal sulcus posterior to the quadrate (usually understood as housing the external auditory meatus) and that this membrane was contacted by a non-ossified extrastapes or a tympanic process of the stapes (Fig 4A and 4B). As already noted by Allin [5] and Allin and Hopson [6], one of the main problems of this theory is the lack of evidence regarding the presence of a connection between the stapes and the purported postquadrate tympanic membrane. A lateral process present in *Scalenodon*, alleged to be an ossified extrastapes, was the main line of evidence provided by the supporters of this theory [27–28, 64]. In Parrington’s [27] view, this process would have projected well beyond the paroccipital process and contacted the tympanic membrane at the inner end of the partial tube formed by the squamosal (Fig 5A). Contrary to this hypothesis, Allin [5] suggested that the purported process observed in *Scalenodon* represents an extension of the usual contact of the stapes and the quadrate based on the analysis of *Exaeretodon*. Allin [5] showed that in this taxon, the quadrate and the entire width of the lateral end of the stapes were in contact precluding the development of an extrastapes such as that proposed for *Scalenodon*. Additionally, he stated that no evidence was to be found that would suggest the presence of a cartilaginous extrastapes in this taxon. Parrington [28] argued against this interpretation stating that it only relied on a single specimen of *Exaeretodon* and omitted the evidence provided by several authors that recognized or hypothesized the presence of an extrastapes in more basal taxa. Additionally, he mentioned that the generalized absence of an extrastapedial process might be due to secondary loss given its delicate nature. The present study shows that none of the many non-mammaliaform cynodonts analyzed (including also basal cynodonts and probainognathids) have an ossified extrastapes or clear evidence of loss of this element by damage. As preserved, the stapes of *Scalenodon* is broken in two pieces and the portion with the putative extrastapes shows evident deformation (recognized from the shape of the stapedial foramen). Therefore we consider this ‘lateral process’ as a consequence of taphonomic distortion (Fig 1F: htp).

A tympanic process has also been interpreted to be present in *Thrinaxodon* by Fourie [30] who recognized a very small process (that he dubbed extrastapes; Fig 5B) on the lateral surface of the stapes and stated that it closely approached the lower end of the squamosal groove housing the external auditory meatus, a fact that seems difficult to accept given the minute nature of this structure. Fourie [30] also suggested that this process might be instead part of the dorsal process and that the extrastapes of Parrington [27] could be what he identified as the quadrate process. In either of these scenarios, the purported tympanic process (extrastapes or quadrate process of Fourie [30]) would have been very small and not comparable to the one described in *Scalenodon* by Parrington [27]. A well-preserved stapes of *Thrinaxodon*, previously described by Estes [29], shows a smooth articulation with the quadrate, and lack any of the processes discussed by Fourie [30]. Our analysis on the stapes of *Thrinaxodon*, to be expanded in a forthcoming contribution, shows that similar to the cynodonts analyzed here, the lateral surface of the stapes contacted the quadrate, in agreement with Estes [29]. Thus, both the tympanic and the quadrate processes described by Fourie [30] would have been part of the stapes-quadrate contact surface.

Additionally, in accordance with the theory suggesting a postquadrate tympanic membrane and a cartilaginous extrastapedial process, some authors [25, 27] stated that non-mammaliaform cynodonts had a restricted stapes-quadrate contact. Under this view, it was the anterior portion of the lateral end of stapes the one that was in contact with the quadrate whereas the posterior half of the distal end of the stapes lay posterior to the quadrate and faced freely laterally. In this context, a mechanical role was associated with the anterior crus which, together with the expanded stapedial footplate, prevented the excessive medial movement of the ventral end of the mobile quadrate. The free posterior lateral portion of the stapes was thought to be in contact with the paroccipital process or the postquadrate tympanic membrane directly or through a non-ossified extrastapes. Most of these suggestions were based on specimens in which the stapes showed signs of postmortem displacement. This fact makes the unambiguous identification of the stapes contacts with the surrounding structures difficult to ascertain. In many of the specimens surveyed here and elsewhere [5, 43], the stapes and the quadrate are preserved in contact with each other, showing that the whole lateral surface of the stapes contacted the quadrate. Thus, it is clear that in these forms the stapes did not contact the paroccipital process directly. Additionally, we found no evidence on the body of the stapes that would hint the presence of a non-ossified extrastapes in any of the specimens analyzed. These results are in accordance with the early work of Broom [42, p.423] who, although lacking much of the evidence available now, when referring to basal cynodonts stated that ‘there is apparently no extrastapedial, and certainly the greater part of the outer end of the stapes is firmly fixed to the quadrate’.

A second theory proposes that the tympanic membrane was associated with the postdentary bones (Fig 5C). The main advocates of this idea have been Allin [5], Kermack et al. [53], Kermack and Mussett [66], Kermack [67], and Kemp [26]. According to this theory, the sound waves were transmitted through the articular, quadrate, and stapes to the fenestra ovalis. We find this proposal preferable to the former as it does not impose a change in the sound transmitting bone chain from non-mammaliaform cynodonts to mammals. Additionally, it does not require speculating about the presence of a tympanic process of the stapes. The drawback to this view is that the presence of a conspicuous squamosal sulcus remains unexplained.

The third theory proposes a compromise solution for these issues suggesting that a postdentary and a postquadrate tympanic membrane were present (separated or continuous; Fig 5D and 5E). The main supporters of this theory have been Watson [59], Allin [68], Allin and Hopson [6], and Meng et al. [62]. A continuous eardrum from the postquadrate area to the postdentary region (Fig 5D) has been endorsed by modern students accepting the presence of both tympanic membranes [6, 62, 68]. Ontogenetic evidence for such an hypothesis was presented by Meng et al. [62] who, following Presley [8], argued that the developmental pattern of extant mammals (e.g., *Didelphis*) shows the tympanic membrane supported by parts of the second arch cartilage (i.e., the tympanohyal and the element of Spence) prior to being completely supported by the tympanic. However, the problem with accepting this view is that there is no extant functional analogue with a comparable morphology in a post-hatching stage. On the other hand, the theory that suggests the presence of two non-continuous eardrums (Fig 5E) with the ultimate loss of the postquadrate eardrum would be more in line with the evidence available from adult extant mammals. The main drawback of the latter hypothesis is that it implies that the tympanic membrane of sauropsids and more basal tetrapods (also early synapsids if they are reconstructed as presenting a postquadrate tympanum) cannot be homologized with that of extant mammals (and the postdentary tympanic membrane of more basal cynodonts) as suggested previously [63, 69].

One additional problem with those hypotheses reconstructing a postquadrate tympanic membrane is whether it was in contact to the stapes or, as the available evidence suggests, was not connected to it (see above) [62]. Following Allin [68], Allin and Hopson [6] stated that although there is little evidence of a tympanic process in non-mammaliaform cynodonts, a connection between the stapes and the postquadrate tympanic membrane may have persisted in forms with a stapedial dorsal process. Nevertheless, Allin [68] did not mention the dorsal process as participating in such a link but pointed to a facet or bump on the posterolateral angle of the stapes. The suggestion by Allin and Hopson [6] implies a homology problem as it is not the dorsal process of the stapes but the tympanic process that is related to the contact with the eardrum as a cartilaginous extrastapes in extant sauropsids with tympanic ears and, purportedly, in more basal synapsids as they also acknowledge [6]. On the other hand, the dorsal process is associated with the connection between stapes and paroccipital process (and maybe to the anterior hyoid cornu). Additionally, in non-mammaliaform cynodonts, the observed dorsal process is a triangular-shaped lamina that points dorsally or dorsomedially from the dorsolateral portion of the posterior crus (except for some specimens of *Thrinaxodon* in which the dorsal process is more robust and developed from the lateral platform). This morphology is not suggestive of the attachment of a cartilaginous process; in such a case, a posteriorly or posterolaterally oriented process would be expected. The characteristics of the dorsal process recognized in non-mammaliaform cynodonts are compatible to the insertion of a small ligament or muscle such as Paauw’s cartilage or the stapedial muscle [6, 23, 31]. In humans, the stapedial muscle dampens the vibrations of the stapes and links this bone to the posterior wall of the tympanic cavity [70]. Thus we propose that the stapedial muscle connected the stapes to the paroccipital process in basal cynodonts and that this muscle attached to the dorsal process when present (Fig 5C and 5D). Other muscular insertions on the stapes of basal cynodonts such as that hypothesized by Kemp [25] on the ventral surface of the stapes seem unlikely. Even if a link between the dorsal process and the postquadrate eardrum is accepted, an additional problem is that the dorsal process of the stapes is not ubiquitous among non-mammaliaform cynodonts. Furthermore, we have registered intraspecific variation of this feature among *Massetognathus* specimens. A stapes-postquadrate tympanum connection through the dorsal process would not be possible in forms without such a structure as no other attachment site for the extrastapedial portion has been recognized. These two scenarios (i.e., either the postdentary or both tympanic membranes related to the stapes) probably implied very different hearing capabilities, a fact that we consider doubtful among representatives of the same species. Hence, in our view, the presence of a dorsal process cannot be used to infer a connection between the stapes and the postquadrate tympanic membrane.

It has been stated that a functional postquadrate eardrum could have been present in non-mammaliaform cynodonts even in the absence of a stapedial-tympanic membrane direct connection [62]; however, we regard this argument as difficult to test. Based on the evidence at hand, we adopt a conservative posture and propose that the squamosal sulcus and the postquadrate tympanic membrane (if present) in non-mammaliaform cynodonts should be thought as relictual and not linked to the stapes. In these forms, it is not evident that the hypothetical postquadrate tympanic membrane participated directly in the sound transmission (Fig 5E).

## Conclusions

Contrary to the established view of a conservative stapedial morphology among non-mammaliaform cynodonts, our results highlight the presence of intra and interspecific variation of this bone. We suggest that the intraspecific variation of the stapedial morphology that cannot be associated with different ontogenetic stages may hint the existence of cryptic species. There have been recognized differences in the relative size and curvature of the crura, the presence of medial and lateral platforms, the presence of anterior and posterior projections, the presence of a dorsal process, the relative size of the stapedial foramen, and the dorsoventral extension of the stapedial footplate. The inclusion of stapedial features as phylogenetic characters in a total evidence data matrix of Gomphodontia (including cranial, postcranial, and dental characters), showed that some of them are phylogenetically informative.

In our view, there was a functional postdentary tympanic membrane. We accept that a relictual postquadrate tympanic membrane associated with the squamosal sulcus may have been present. On the other hand, a link between the postquadrate eardrum (if present) and the stapes is not supported by our observations. There is no evidence to suggest the presence of a non-ossified extrastapes. The dorsal process of the stapes observed in some taxa is interpreted as the site for attachment of the stapedial muscle; and it cannot be readily homologized with the tympanic process of other taxa.

## Supporting Information

S1 Datamatrix Character-taxon data matrix. (MESQUITE)
(ZIP)

S2 Datamatrix Character-taxon data matrix. (TNT)
(ZIP)

S1 Fig Most parsimonious trees obtained from the unconstrained search. All taxa included. Letters at the nodes indicate high-level clades: C, Cynognathia; G, Gomphodontia; T, Traversodontidae. (TIFF)
(TIF)

S2 Fig Most parsimonious trees obtained from the restricted analysis excluding *Nanogomphodon*. Letters at the nodes indicate high-level clades: C, Cynognathia; G, Gomphodontia; T, Traversodontidae. (TIFF)
(TIF)

S3 Fig Strict consensus tree obtained from the unconstrained search. All taxa included. Letters at the nodes indicate high-level clades: C, Cynognathia; G, Gomphodontia; T, Traversodontidae. (TIFF)
(TIF)

S4 Fig Strict consensus tree of the constricted search forcing the monophyly of trirachodontids. All taxa included. Letters at the nodes indicate high-level clades: C, Cynognathia; G, Gomphodontia; T, Traversodontidae; Tr, Trirachodontidae. (TIFF)
(TIF)

S5 Fig Strict consensus tree of the constricted search forcing the monophyly of trirachodontids and excluding *Nanogomphodon*. All taxa included. Letters at the nodes indicate high-level clades: C, Cynognathia; G, Gomphodontia; T, Traversodontidae; Tr, Trirachodontidae. (TIFF)
(TIF)
