# Supplementary figures and images for: The Stapes of Gomphodont Cynodonts: Insights into the Middle Ear Structure of Non-Mammaliaform Cynodonts
Source: PLoS One. 2015 Jul 15;10(7):e0131174. doi: 10.1371/journal.pone.0131174 (PMC4503721; doi:10.1371/journal.pone.0131174)

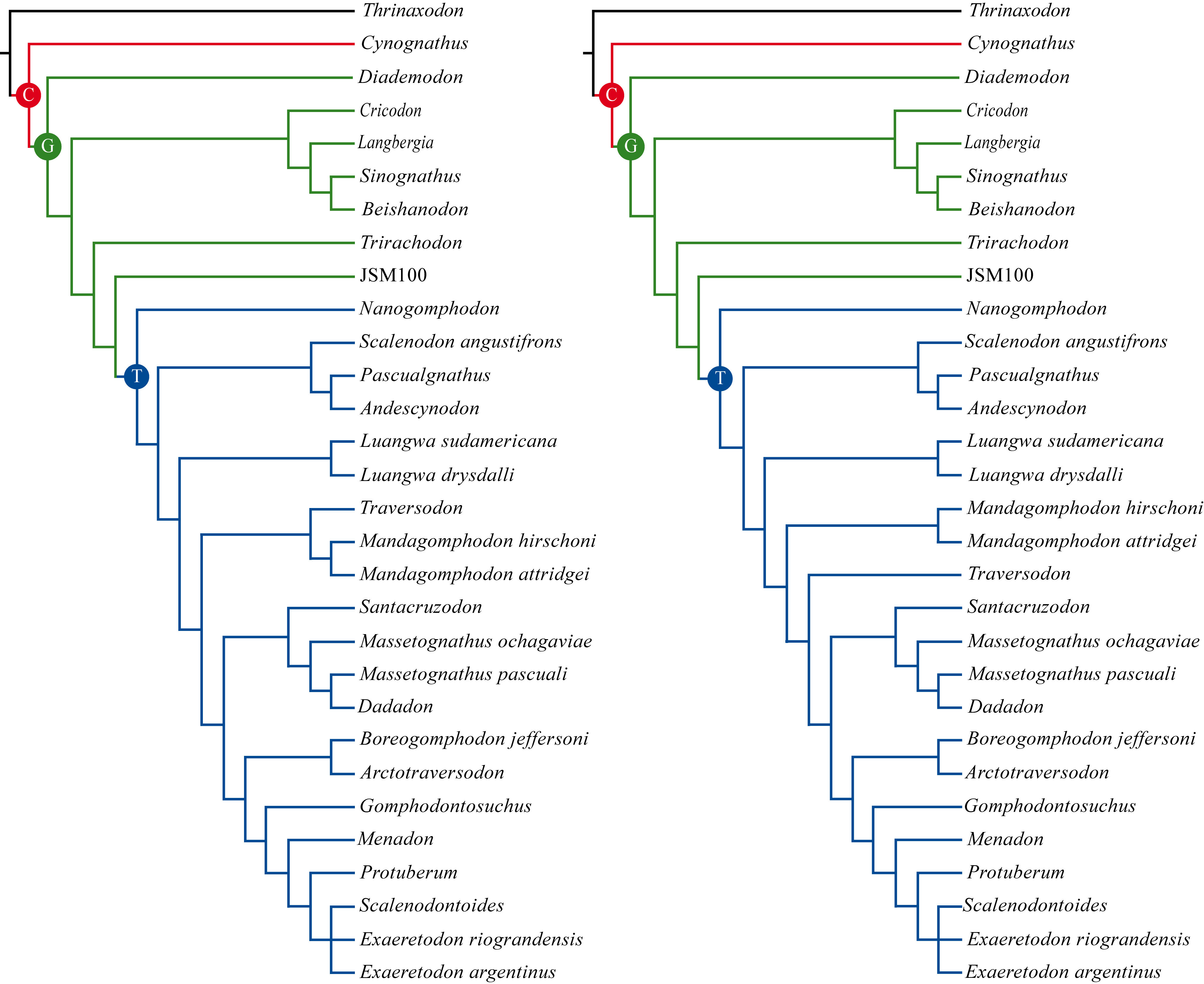

Supplement: S1 Fig — All taxa included. Letters at the nodes indicate high-level clades: C, Cynognathia; G, Gomphodontia; T, Traversodontidae. (TIFF) (TIF) [file pone.0131174.s003.tif]

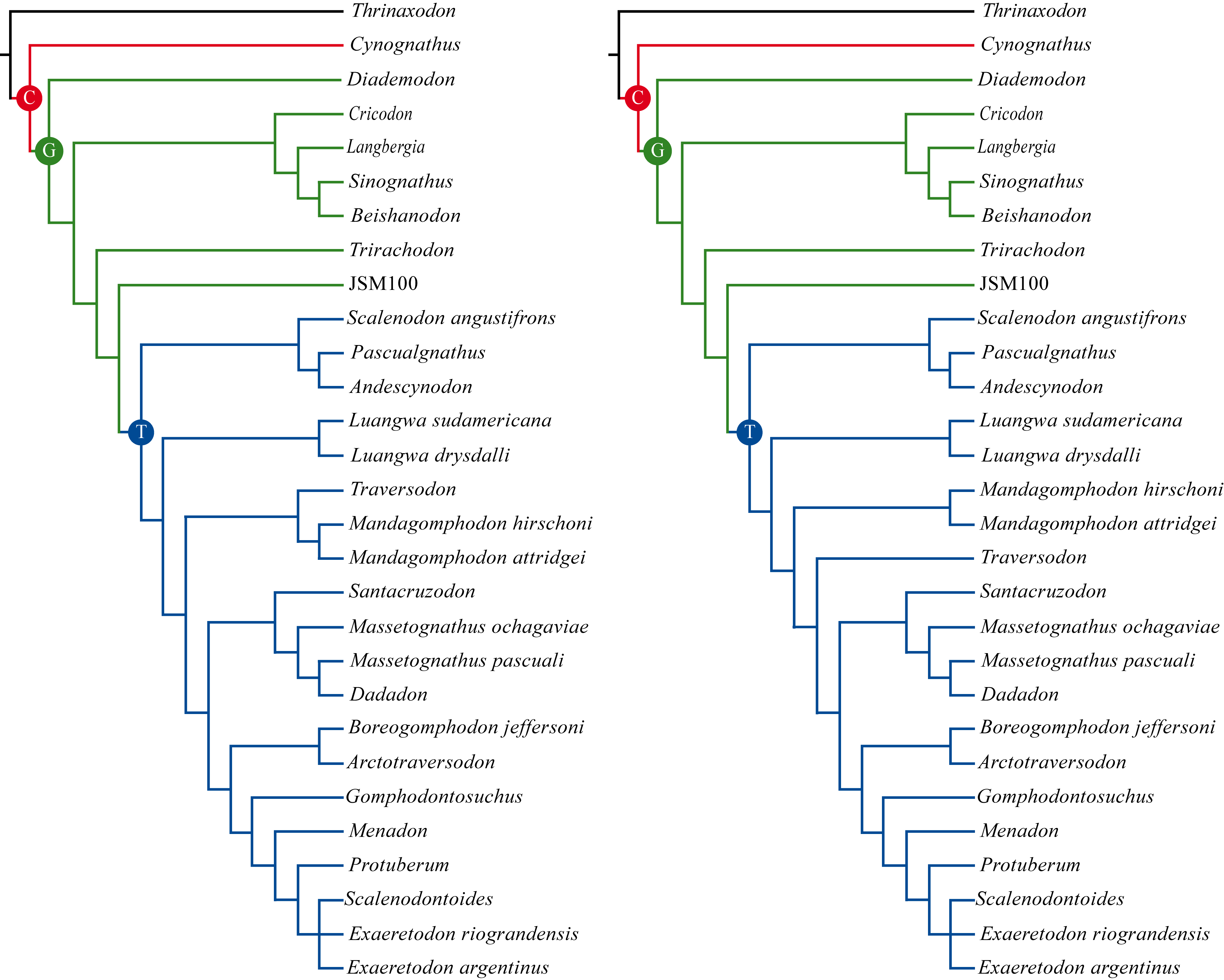

Supplement: S2 Fig — Letters at the nodes indicate high-level clades: C, Cynognathia; G, Gomphodontia; T, Traversodontidae. (TIFF) (TIF) [file pone.0131174.s004.tif]

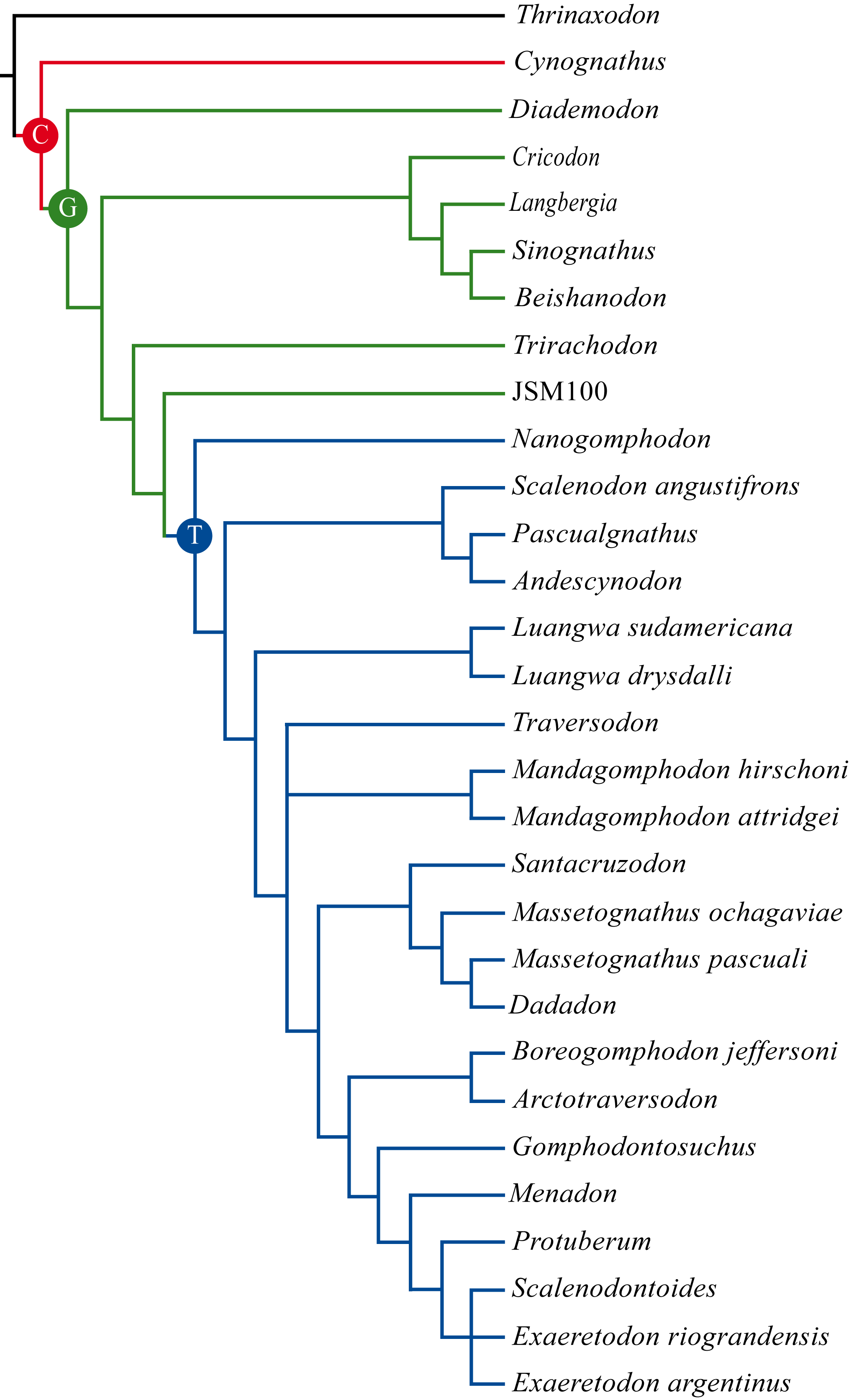

Supplement: S3 Fig — All taxa included. Letters at the nodes indicate high-level clades: C, Cynognathia; G, Gomphodontia; T, Traversodontidae. (TIFF) (TIF) [file pone.0131174.s005.tif]

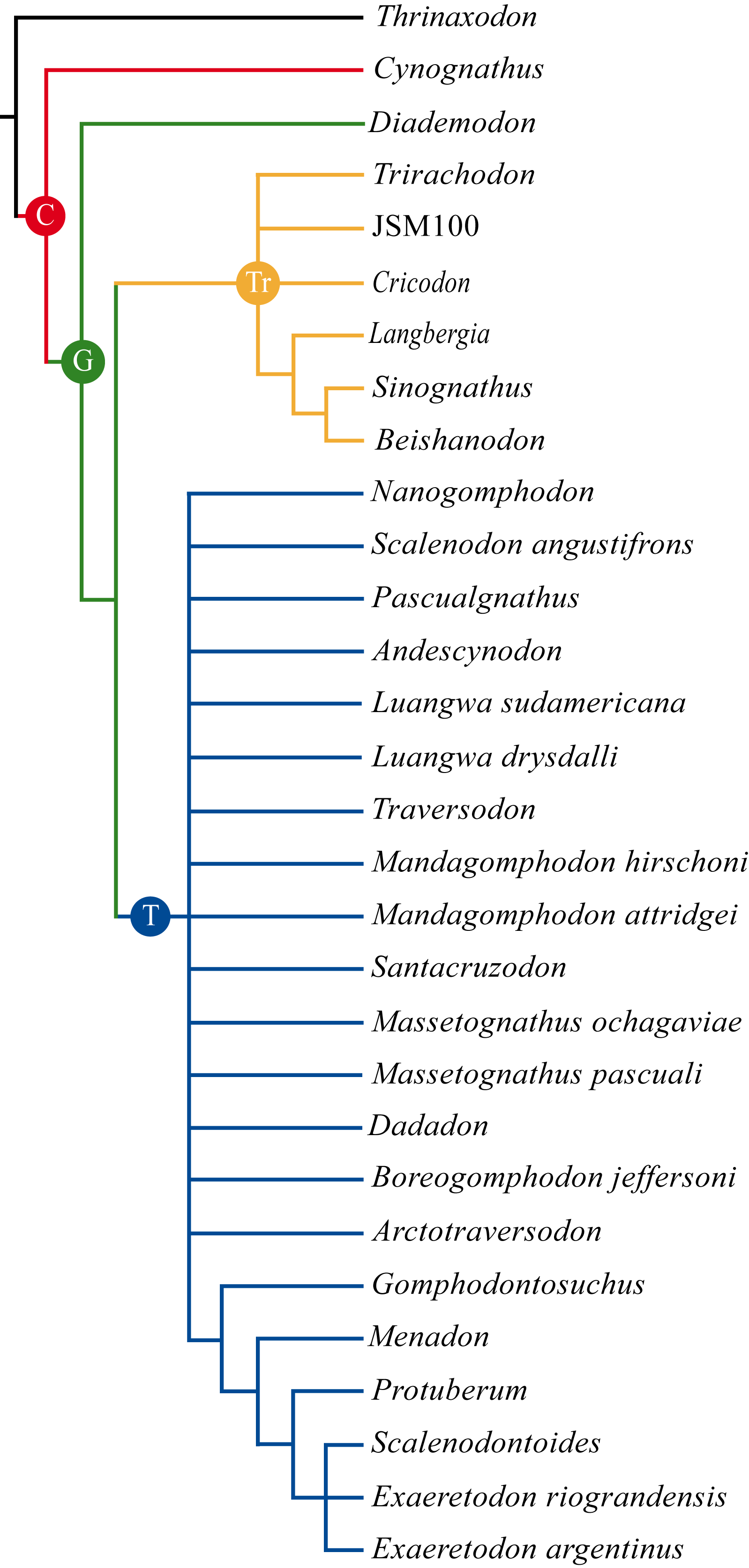

Supplement: S4 Fig — All taxa included. Letters at the nodes indicate high-level clades: C, Cynognathia; G, Gomphodontia; T, Traversodontidae; Tr, Trirachodontidae. (TIFF) (TIF) [file pone.0131174.s006.tif]

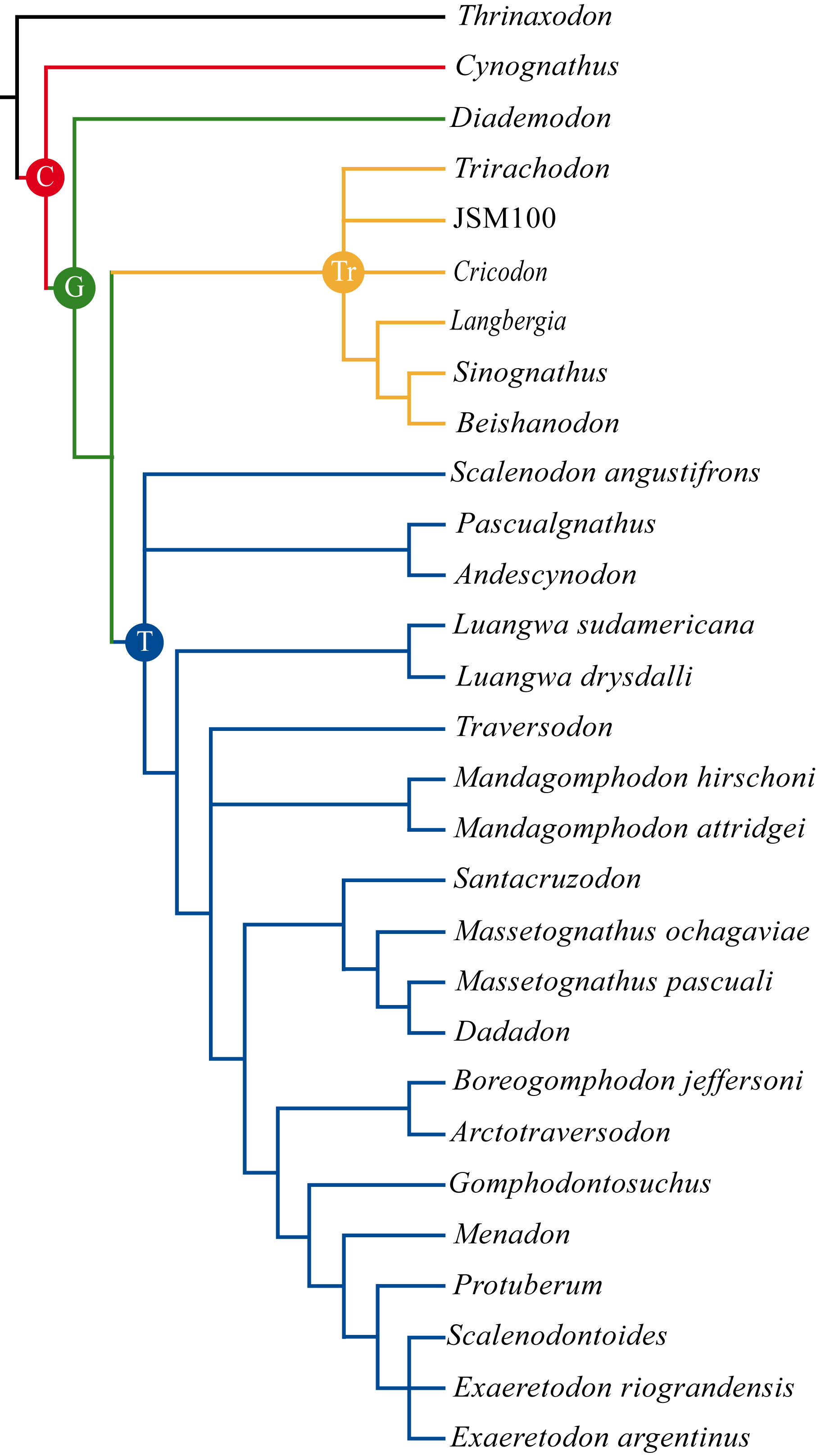

Supplement: S5 Fig — All taxa included. Letters at the nodes indicate high-level clades: C, Cynognathia; G, Gomphodontia; T, Traversodontidae; Tr, Trirachodontidae. (TIFF) (TIF) [file pone.0131174.s007.tif]
